# Estrogens and Antioxidants Prevent the Formation of Tubular Aggregates in Aging Male Mice

**DOI:** 10.3390/ijms26189122

**Published:** 2025-09-18

**Authors:** Giorgia Rastelli, Matteo Serano, Barbara Girolami, Alice Brasile, Vincenzo Sorrentino, Laura Pietrangelo, Feliciano Protasi

**Affiliations:** 1 CAST—Center for Advanced Studies and Technology, University G. d’Annunzio of Chieti-Pescara, I-66100 Chieti, Italy; giorgia.rastelli@unich.it (G.R.); barbara.girolami@unich.it (B.G.); alice.brasile@unich.it (A.B.); feliciano.protasi@unich.it (F.P.); 2 DNICS—Department of Neuroscience, Imaging and Clinical Sciences, University G. d’Annunzio of Chieti-Pescara, I-66100 Chieti, Italy; 3 Molecular Medicine Section, Department of Molecular and Developmental Medicine, University of Siena, I-53100 Siena, Italy; matteo.serano@unisi.it (M.S.); vincenzo.sorrentino@unisi.it (V.S.); 4 DMSI—Department of Medicine and Aging Sciences, University G. d’Annunzio of Chieti-Pescara, I-66100 Chieti, Italy

**Keywords:** skeletal muscle, electron microscopy, store-operated calcium entry, sarcoplasmic reticulum

## Abstract

Tubular aggregates (TAs), ordered arrays of sarcoplasmic reticulum (SR) tubes, are the main morphological alteration found in muscle biopsies from patients affected by TA myopathy (TAM). TAM has been linked to mutations in the genes encoding for STIM1 and ORAI1, which are two proteins that mediate Store-Operated Ca^2+^ entry (SOCE). SOCE is a mechanism that allows recovery of extracellular Ca^2+^ during fatigue, when the SR becomes depleted. As TAs also form in fast-twitch muscle fibers of aging male mice (not in females), we studied the effect of sex hormones on the aggregation of TAs during aging. We administered estrogen (ad libitum in drinking water) to male mice from 10 to 18 months of age and then evaluated the following: (a) the presence of TAs using histology and electron microscopy (EM); (b) oxidative stress, a mechanism that could underlie damage to proteins and membranes (and possibly their accumulation in TAs); and (c) SOCE function during ex vivo stimulation in the presence or absence of external Ca^2+^ or SOCE blocker (BTP-2). The results collected indicate that treatment with estrogen (a) significantly reduced the formation of TAs; (b) reduced oxidative stress, which was elevated in aging male mice; and (c) restored SOCE, i.e., the capability of aged EDL muscles to use external Ca^2+^ by promoting maintenance of Ca^2+^ Entry Units (CEUs, the intracellular junctions that mediate SOCE). Finally, we also show that formation of TAs is reduced by treatment of mice with N-acetilcysteine (NAC), a potent antioxidant also administered ad libitum in drinking water.

## 1. Introduction

Tubular aggregates (TAs), ordered arrays of straight membrane tubes found in different myopathies, are the main morphological alteration found in biopsies of patients affected by TA myopathy (TAM). The clinical spectrum of TAM varies from asymptomatic to slowly progressive limb weakness, muscle pain and cramping, joint deformities in the arms and legs [1,2,3], and finally, Stormorken syndrome, the most severe form of the disease with multisystemic signs [4]. TAM has been predominantly linked to mutations in the genes encoding (i) stromal interaction molecule-1 (STIM1) and (ii) calcium release-activated calcium (CRAC) channel ORAI1, which are two proteins that mediate a mechanism known as Store-Operated Ca^2+^ entry (SOCE) [3,5,6,7,8,9,10]. In addition, in a smaller fraction of patients, TAM has been linked to mutations in the gene encoding calsequestrin-1 (CASQ-1), the major Ca^2+^-binding protein of the sarcoplasmic reticulum (SR) [7,11,12], and in patients carrying mutations of *RYR1*, the gene encoding the skeletal muscle isoform of the SR Ca^2+^ release channel [13]. The SR represents the main intracellular Ca^2+^ store in skeletal muscle fibers and is structurally organized to sustain the release and uptake of Ca^2+^ during repeated cycles of contraction [14,15]. SOCE is a pathway that allows recovery of external Ca^2+^ when intracellular stores are depleted [10,16,17,18,19,20].

First reported in 2001 [21], SOCE is a relatively new phenomenon in skeletal muscle [22,23], where it allows the recovery of extracellular Ca^2+^ during fatigue [21,24]. Also, muscle SOCE is mediated by the interaction between STIM1, localized in the SR membrane, and ORAI1, located in the transverse tubules (TTs) [25,26]. In muscle, SOCE [27,28] was also proposed to contribute to (i) muscle growth during maturation [29,30] and (ii) muscle disease [27,31]. We recently discovered that acute exercise promotes remodeling of the sarcotubular system (SR and TTs), leading to the assembly of new structures named Ca^2+^ Entry Units (CEUs). These specialized intracellular junctions form through the association between SR and TTs at the I band of sarcomeres. The formation of CEUs increases the co-localization of ORAI1 with STIM1, thereby providing the structural base for activation of SOCE [32]. Indeed, we demonstrated that these new junctions provide a preferential pathway for recovering Ca^2+^ from the extracellular space (and enhance SR refilling) during repetitive muscle activity [33,34,35].

TAs, besides being found in muscle fibers of TAM patients, are also present in the skeletal muscle of aged wild-type (WT) mice [36,37]. TAs in mice stained positive for CASQ1 [36], the main Ca^2+^ binding protein of the SR [38], which modulates the function of the ryanodine receptor (RYR) during excitation–contraction (EC) coupling [39,40,41,42,43]. CASQ1 has been proposed also to modulate the SOCE function [44,45]. Boncompagni and colleagues showed that TAs in aging mice do not contain TTs and are negative for RYR staining [36]. We have recently shown (i) that STIM1 and ORAI1 accumulate in the TAs of *extensor digitorum longus* (EDL) muscle fibers of aging mice, and that SOCE is dysfunctional in muscle containing TAs; (ii) 1 year of voluntary in-wheel cage exercises (from 12 to 24 months of age) significantly reduced the formation of TAs, and promoted the maintenance of CEUs and SOCE function [46].

As TAs during aging are found only in male mice but not in females [8,36,37,47], in the current study, we tested the effect of sex hormones on the accumulation of TAs and on SOCE function in the EDL muscle of WT male mice. We analyzed muscles from three groups of animals: adult controls (10 months old, referred to as 10 mo.); aging mice (18 mo.); and mice treated with 17-β-Estradiol from 10 to 18 months of age. A combination of structural (light and electron microscopy) and functional (ex vivo muscle contractility) approaches was used. To investigate mechanisms potentially responsible for the improper accumulation of proteins and membranes in TAs, we tested the hypothesis that the formation of TAs during aging may be mediated by increased oxidative stress. First, we verified that oxidative stress was reduced in mice treated with estrogen, and then administered N-acetylcysteine (NAC), a potent antioxidant, provided ad libitum in drinking water from 10 to 18 months of age, verifying whether this drinking regimen could reduce the assembly of TAs.

## 2. Results

### 2.1. Estrogen Prevented Formation of TAs

**Figure 1:**
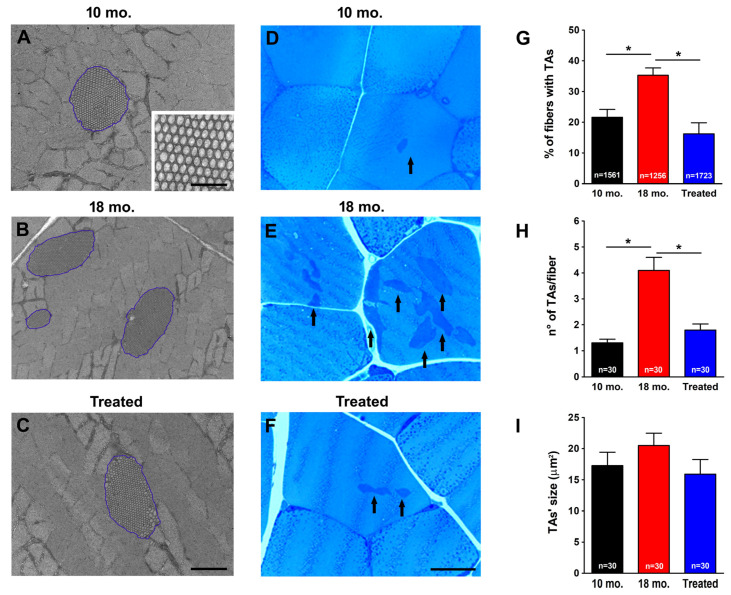
**Histological and quantitative EM analysis of the incidence of TAs in EDL fibers.** (**A**–**C**) Representative EM images from transverse sections of EDL muscles from 10 mo. (**A**), 18 mo. (**B**), and 18 mo. estrogen-treated (**C**) WT male mice: TAs are outlined by the blue line, and the enlarged detail in (**A**) shows the ordered array of SR tubes. (**D**–**F**) Representative histological images of transverse sections from the EDL muscle taken from 10 mo. (**D**), 18 mo. (**E**), and 18 mo. estrogen-treated (**F**) WT male mice. TAs are represented by black arrows. (**G**–**I**) Bar plots show the quantitative analyses of the percentage of EDL fibers containing TAs (**G**), the number of TAs per fiber (**H**), and the average size of individual TAs, respectively (**I**). Data in (**G**–**I**) are shown as mean ± SEM; * *p* < 0.05; *n* = number of fibers analyzed from 10 mo. (3 mice), 18 mo. (3 mice), and 18 mo. estrogen-treated (3 mice) WT male mice. Scale bars: (**A**–**C**) = 2 µm; (**D**–**F**) = 20 μm; inset in (**A**) = 300 nm.

TAs are clearly visible in electron microscopy (EM) images as arrays of SR tubes. In Figure 1A–C, TAs are outlined by the blue line in samples from 10 mo. (Figure 1A), 18 mo. (Figure 1B), and from 18 mo. estrogen-treated (Figure 1C) WT male mice: the enlarged detail in Figure 1A shows the intrinsic order of tubes in the array. Using histological images taken from transversal sections of EDL muscles (Figure 1D–F), we evaluated (i) the percentage of fibers containing TAs (Figure 1G); (ii) the number of TAs per fiber (Figure 1H); and (iii) the average size of TAs (Figure 1I).

Quantitative analysis of the percentage of fibers containing TAs in histology indicated that, while TAs accumulate with increasing age from 10 to 18 months of age, estrogen treatment is effective at preventing their formation during aging, as the percentage of fibers containing TAs was reduced from ∼35% (in 18 mo. mice) to ∼16% following treatment with estrogen (Figure 1G). We then measured the number of TAs per fiber and their average size in EM and found that treatment with estrogen was able to reduce both (i) the average number of TAs per fiber (from 4.5 to 1.9 in 18 mo. vs. 18 mo. estrogen-treated mice, respectively; Figure 1H), and (ii) the average size of the few remaining TAs (from 22 to 16 μm^2^ in 18 mo. vs. 18 mo. estrogen-treated mice, respectively; Figure 1I).

### 2.2. Estrogen Maintained Extracellular Ca^2+^ Dependence and Cross-Sectional Area (CSA) of Muscle Fibers

**Figure 2:**
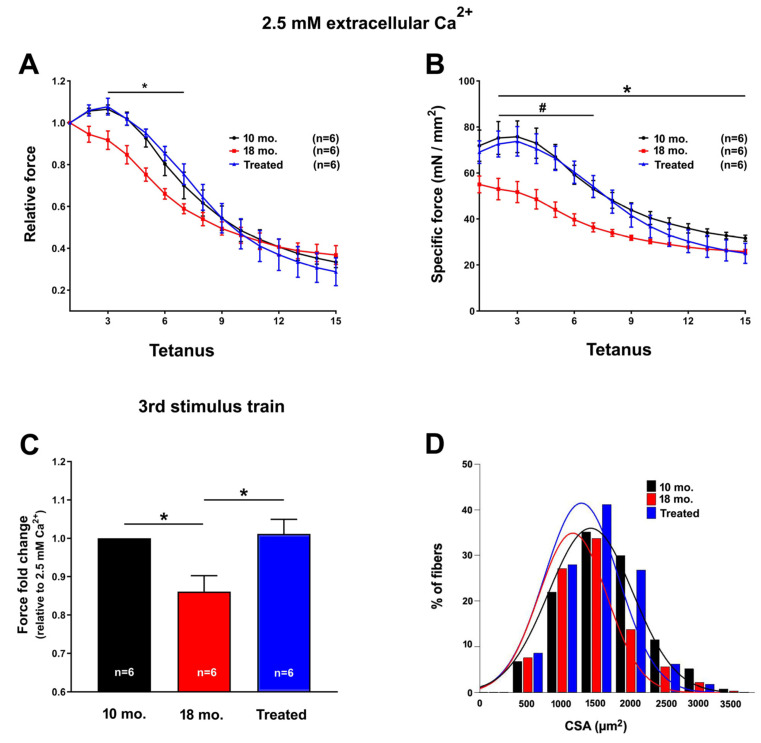
**Ex vivo fatigue protocol in EDL muscles and cross-sectional area (CSA) of fibers assessed using light microscopy.** (**A**,**B**) Time course of relative (**A**) and specific (**B**) force decay (normalized to the first stimulus train) during 30 consecutive stimulus trains (60 Hz with 1 s duration every 5 s) in the presence of a standard KH solution containing 2.5 mM Ca^2+^. The asterisks in (**A**,**B**) indicate the window of significant statistical difference between 18 mo. mice (control) vs. the other two groups of mice (10 mo. and 18 mo. estrogen-treated mice). (**C**) Bar plot showing the fold change in force, relative to 10 mo. mice, calculated at the 3rd stimulus train (empty arrow). Data are shown as mean ± SEM; * *p* < 0.05 10 mo. vs. 18 mo.; ^#^ *p* < 0.05 Treated vs. 18 mo. *n* = EDL muscles tested from 10 mo. (from 6 mice), 18 mo. (from 6 mice), and 18 mo. estrogen-treated (from 6 mice) WT male mice. (**D**) Bar plots showing the distribution frequency of CSA evaluated from transversal sections of EDL muscle fibers, stained with Toluidine blue, from 10 mo., 18 mo. old, and 18 mo. estrogen-treated mice (i.e., estrogen from 10 to 18 months of age). Data are shown as mean values.

**Figure 3:**
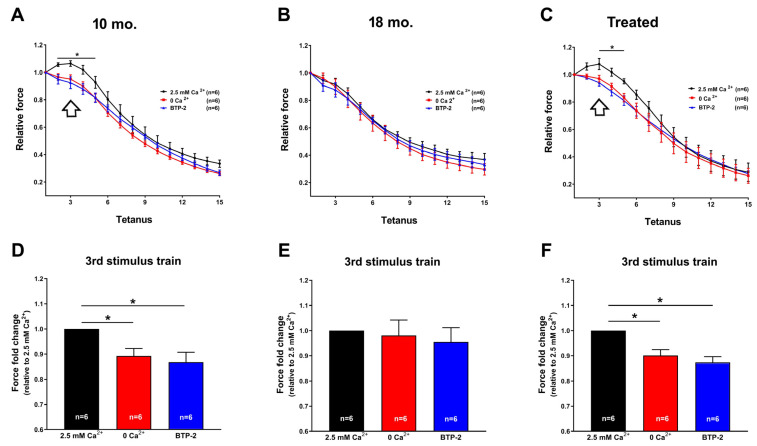
**Ex vivo fatigue protocols in EDL muscles in the presence or absence of extracellular Ca^2+^.** (**A**–**C**) Time course of relative force decay (normalized to the first stimulus train) during a series of 30 repeated stimulations (60 Hz with 1 s duration every 5 s) in the presence of standard KH solution, either containing 2.5 mM Ca^2+^ (black trace), a nominally Ca^2+^-free solution (red trace), or standard KH solution containing 2.5 mM Ca^2+^ supplemented with 10 μM BTP-2 (blue trace). The asterisks in (**A**,**C**) indicate the interval of significant statistical difference between the control condition (2.5 mM Ca^2+^) and the other two conditions (0 Ca^2+^ and presence of BTP2). (**D**–**F**) Histograms showing the fold change in force, relative to the 2.5 mM Ca^2+^ condition, calculated at the 3rd stimulus train (empty arrow). Data are shown as mean ± SEM; * *p* < 0.05; *n* = EDL muscles tested from 10 mo. (6 mice), 18 mo. (6 mice), and 18 mo. estrogen-treated (6 mice) WT male mice.

In ex vivo fatigue experiments, intact EDL muscles dissected from 10 mo., 18 mo., and 18 mo. estrogen-treated mice were subjected to a protocol of 30 consecutive 1 s long, 60 Hz stimulus trains applied every 5 s. The experiments were conducted using a standard KH solution containing 2.5 mM Ca^2+^ (Figure 2) or under conditions designed to reduce/block Ca^2+^ entry, such as a nominally Ca^2+^-free KH solution or standard KH solution supplemented with 10 μM BTP-2 (Figure 3).

When exposed to the standard KH solution containing 2.5 mM Ca^2+^, EDL muscles from 18 mo. mice exhibited both a reduced specific and relative force compared to those observed in muscles from 10 mo. mice (Figure 2A,B). Quantitative analysis of the force fold change relative to the force generated by muscles from 10 mo. mice (Figure 2C), evaluated during the third stimulus, showed a 30% force decay in muscles from 18 mo. mice. Following 8 months of estrogen treatment, EDL muscles from 18 mo. estrogen-treated mice showed a fully restored ability to maintain the contractile force (Figure 2A,B), as the average force fold change was not significantly different from that of 10 mo. animals (Figure 2C).

As force may be influenced by the size of fibers, we measured the CSA of EDL muscle fibers. Compared to the distribution frequency obtained from 10 mo. mice, the fiber CSA profile of the EDL muscles dissected from 18 mo. mice showed a leftward shift in the distribution toward smaller sizes (1000–1500 μm^2^), which is a typical sign of atrophy, whilst the fiber CSA profile of EDL muscles dissected from 18 mo. estrogen-treated mice was partially maintained or restored, as suggested by the distribution pattern of fiber CSA, which was more similar to that of 10 mo. mice (Figure 2D).

To complete ex vivo fatigue experiments, intact EDL musclesdissected from 10 mo., 18 mo., and 18 mo. estrogen-treated mice were subjected to a protocol of 30 consecutive 1 s long, 60 Hz stimulus trains applied every 5 s under conditions designed to eliminate Ca^2+^ entry (Figure 3). Two different approaches were employed to reduce/block Ca^2+^ entry during the fatigue protocol, as depicted in Figure 3: (a) in one series of experiments, muscles were exposed to a nominally Ca^2+^-free KH solution, where Ca^2+^ was substituted with an equimolar concentration of Mg^2+^; (b) in the second series of experiments, muscles were exposed to a standard KH solution supplemented with 10 μM BTP-2. As seen in previous studies conducted at 4 months of age [32,33,34], these interventions also had a modest yet statistically significant impact on force production during repetitive stimulation in EDL muscles from 10 mo. WT mice (Figure 3A). However, under conditions where Ca^2+^ entry was limited or blocked (e.g., when external Ca^2+^ was removed or in the presence of BTP-2), EDL muscles from 18 mo. mice did not exhibit any significant reduction in force generation compared to the standard condition (Figure 3B). This suggests that, unlike younger muscles, aged fibers are unable to recruit additional extracellular Ca^2+^ during the early phase of the fatigue protocol i.e., the *bump phase* present in EDLs from 10 mo. old mice while missing in EDLs from 18 mo. old mice. The *bump phase* indicates a potentiation of contraction due to the activation of Ca^2+^ entry [34]. Nevertheless, it is important to note that 18 mo. muscles still contract in the presence of external Ca^2+^ (as shown in Figure 2), indicating that the machinery required to respond to Ca^2+^ is fully functional. Conversely, muscles from 18 mo. estrogen-treated mice (such as those from 10 mo. mice) did show the potentiation of contraction in the presence of external Ca^2+^ (*bump phase*), indicating the ability to use external Ca^2+^ under fatigue conditions, and showed a modest, but statistically significant, decrease in contractile force when Ca^2+^ influx was reduced/prevented (Figure 3C).

Quantitative examination of the decline in force fold change, assessed during the third stimulus train, indicated that EDL muscles from 10 mo. and 18 mo. estrogen-treated mice experienced a decrease in force of 20–25% when Ca^2+^ entry was reduced/blocked (Figure 3D,F) compared to conditions where Ca^2+^ entry was allowed. Conversely, there was no notable variance in contractile force observed in muscles from 18 mo. mice, whether external Ca^2+^ was present or absent (Figure 3E).

### 2.3. Estrogen Treatment Counteracted the Age-Related Decline in Structural Components of Ca^2+^ Entry Units (CEUs)

**Figure 4:**
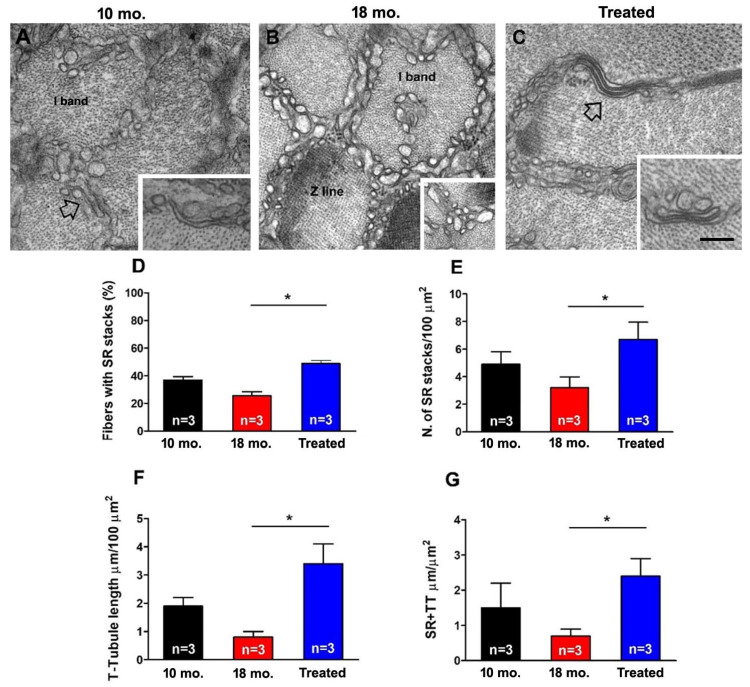
**Electron micrographs and quantitative analysis of CEUs.** (**A**–**C**) Organization of the SR at the I band in 10 mo. (**A**), 18 mo. (**B**), and 18 mo. estrogen-treated (**C**) WT male mice. The inset in (**B**) shows the organization of SR at the I band of sarcomere, and the empty arrows and the insets in (**A**–**C**) show SR stacks. (**D**,**E**) Percentage of fibers containing SR stacks and number of SR stacks/100 μm^2^ in the transverse section. (**F**,**G**) Extension of TTs at the I band in 100 μm^2^ of the transverse section and extension of SR-TT contacts at the I band. Data are shown as mean ± SEM; * *p* < 0.05; *n* = EDL muscles analyzed from 10 mo. (3 mice), 18 mo. (3 mice), and 18 mo. estrogen-treated (3 mice) WT male mice. Scale bar (**A**–**C**) = 0.1 μm; insets = 0.2 μm.

In skeletal muscle fibers, CEUs represent intracellular junctions that allow functional coupling between STIM1 and ORAI1 to promote Ca^2+^ entry during SOCE, thus limiting fatigue [32,33]. In recent papers, we also described how STIM1 and ORAI1, trapped in TAs, are not functional for SOCE [46]. As EDL muscles from 18 mo. treated mice exhibited a recovered capability to use external Ca^2+^ during repetitive stimulation when compared to age-matched untreated mice (Figure 2 and Figure 3), we assessed and quantified the presence of the structural components needed for the assembly of CEUs, i.e., SR stacks and TT extension at the I band (Figure 4A–C).

We evaluated in transverse section for EM SR stacks and TT extensions at the I band of EDL fibers: (a) SR stacks were less numerous in muscles of 18 mo. mice than those observed in muscles from 10 mo. animals; (b) the TT network within the I band was less present at 18 months of age than in muscle fibers of 10 mo. mice (Figure 4A–C). However, after treatment with estrogen, (i) the incidence of stacks of SR membranes (Figure 4D), (ii) the number of SR-stacks/100 µm^2^ (Figure 4E), (iii) the extent of the TT network at the I band, and finally, (iv) SR/TT contact length (Figure 4G; a parameter that specifically indicates the functional association between TT extensions and flat-parallel stacks of SR within the I band), were all significantly increased in 18 mo. treated mice compared to values in age-matched untreated mice, which were similar or higher than those of 10 mo. animals.

### 2.4. Estrogen Reduced Expression Levels of SOD1, SOD2, and Catalase

**Figure 5:**
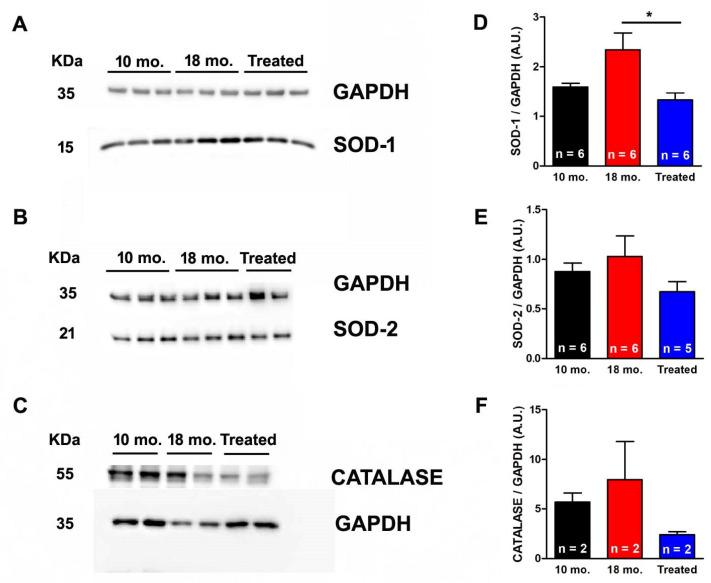
**Levels of copper/zinc (SOD-1), manganese (SOD-2) superoxide dismutase, and catalase in EDL muscle homogenates.** (**A**–**C**) Representative immunoblots showing levels of SOD-1, SOD-2, and catalase in EDL muscle homogenates. (**D**–**F**) Relative band densities normalized to GAPDH of SOD-1, SOD-2, and catalase. Data are shown as mean ± SEM; * *p* < 0.05; *n* = EDL muscle homogenates tested from different 10 mo., 18 mo., and 18 mo. estrogen-treated WT male mice.2.5. NAC Treatment Reduced Formation of TAs.

We tested the hypothesis that the ability of estrogen supplementation to reduce the accumulation of TAs in fast-twitch muscle fibers from male mice is associated with a reduction in oxidative stress levels. We compared oxidative stress in EDL homogenates from mice treated with estrogen versus 10 mo. and 18 mo. untreated mice by measuring the expression levels of (a) copper/zinc superoxide dismutase (SOD-1, the myoplasmic isoform) (Figure 5A,D) and (b) manganese superoxide dismutase (SOD-2, the mitochondrial isoform) (Figure 5B,E), which catalyzes the transformation of superoxide (O^2−^) into oxygen (O_2_) and hydrogen peroxide (H_2_O_2_): the first step for the elimination of reactive oxygen species (ROS). In addition, we also measured expression levels of (c) catalase (Figure 5C,F), the enzymatic scavenger which decomposes H_2_O_2_ into water and molecular oxygen [48,49,50].

Western blot (WB) analyses revealed that, in EDL muscles from 18 mo. untreated mice, SOD-1, SOD-2, and catalase expressions (relative to GAPDH) all increased compared to levels of 10 mo. mice, although this increase was not always statistically significant (Figure 5D–F). However, after estrogen treatment, SOD-1, SOD-2, and catalase expressions (relative to GAPDH) were lowered (Figure 5D–F). In detail, in EDL muscles from estrogen-treated mice, the level of SOD-1 enzyme was significantly reduced by ~43% when compared to 18 mo. untreated mice (Figure 5D). On the other hand, the levels of the mitochondrial isoform of the enzyme (SOD-2) and that of the catalase, although showing a decreasing trend after estrogen treatment, were not significantly reduced.

Using histological and EM images taken from transversal sections of EDL muscles, we verified if treatment with NAC, a potent antioxidant provided to male WT mice ad libitum in drinking water from 10 to 18 months of age [51,52,53], was able to reduce the accrual of TAs, supporting the hypothesis that accumulation of TAs in fast-twitch muscle fibers from male WT mice could be influenced by oxidative stress levels.

**Figure 6:**
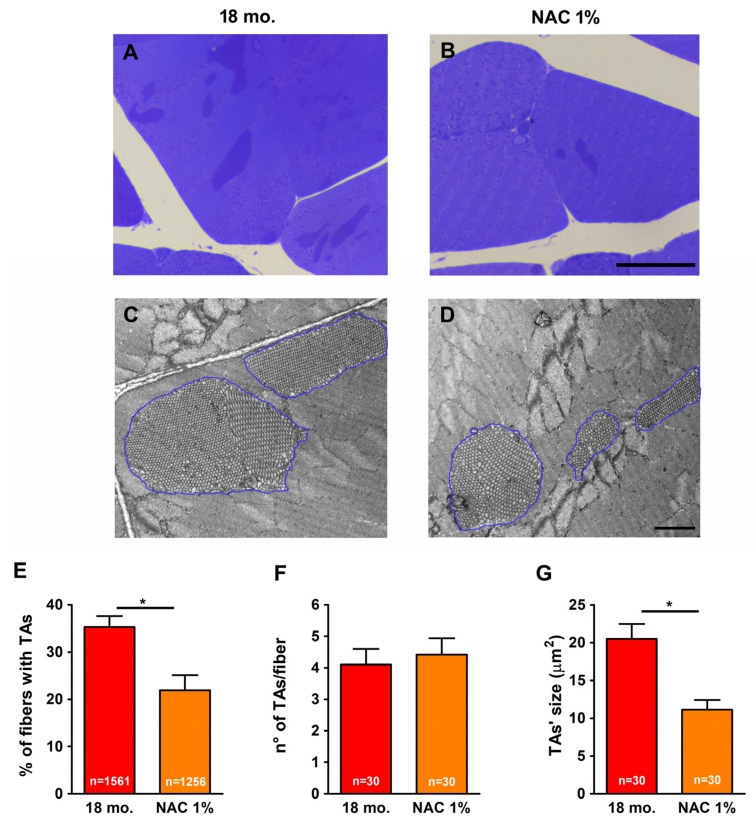
**EM analysis of the incidence of TAs in EDL fibers of NAC-treated mice.** (**A***–***D**) Histological (**A**,**B**) and EM (**C**,**D**) images of TAs in EDL transverse sections from 18 mo. (**A**,**C**), and 18 mo. NAC-treated (**B**,**D**) WT male mice. TAs are outlined by blue lines (**C**,**D**). (**E***–***G**) Bar plots show the percentage of fibers containing TAs; (**E**) the number of fiber TAs/type of fiber; (**F**) and average size of individual TAs (**G**) in 18 mo. and 18 mo. NAC-treated male mice, respectively. Data are shown as mean ± SEM; * *p* < 0.05; *n* = number of fibers analyzed from 18 mo. (3 mice) and 18 mo. NAC-treated (3 mice) WT male mice. Scale bars (**A**,**B**) = 20 μm; (**C**,**D**) = 1 μm.

When compared to age-matched 18 mo. untreated mice, NAC-treated mice showed a significant reduction in (i) the percentage of fibers presenting TAs (Figure 6A), with values similar to that of 10 mo. mice and (ii) the average size of the remaining TAs (Figure 6C). However, no significant variation was observed in the number/fibers of TAs (the fibers in which they were still present) compared to 18 mo. untreated mice (Figure 6B).

## 3. Discussion

### 3.1. The State of the Art

TAs are ordered arrays of SR tubules observed in muscle biopsies from patients affected by different myopathies, and they represent the primary morphological alteration in patients affected by TAM [6,8,16,54,55,56,57,58]. TAs are also found in fast-twitch muscle fibers of male aging WT mice, but not in females [8,36,46,59]. To date, the reason for this gender difference remains unclear.

In 2021, we demonstrated a correlation between the presence of TAs and SOCE dysfunction in the EDL muscles of aging male mice. The presence of TAs in aged EDL fibers was associated with (i) muscle weakness and increased susceptibility to fatigue, and (ii) abnormal accumulation of STIM1 and ORAI1 in TAs [46]. In addition, we showed that long-term regular exercise in wheel cages during aging (a) reduced the accumulation of TAs; (b) promoted maintenance of the membrane elements required for the assembly of functional CEUs; and (c) restored SOCE function. These findings suggest that exercise may be an effective strategy to prevent the formation of TAs and SOCE dysfunction. However, the mechanisms underlying the formation of TAs (and how exercise may prevent it) are unknown.

### 3.2. Main Findings

In the present study, we investigated the role of sex hormones on the aggregation of TAs during aging by treating male mice with estrogen administered in drinking water from 10 to 18 months of age. After treatment, we first evaluated the presence of TAs using histology and EM and then assessed SOCE function during ex vivo stimulation under different conditions (i.e., in the presence or absence of external Ca^2+^ or in the presence of a SOCE blocker, i.e., BTP-2). Finally, we investigated whether oxidative stress could be a mechanism contributing to the accumulation of dysfunctional proteins in TAs.

The results collected in the present manuscript demonstrate that treatment with estrogen (i) reduces age-dependent accumulation of TAs in muscles of male WT mice (Figure 1), and (ii) improves the fatigue resistance of muscles at 18 mo. of age during repetitive stimulation (Figure 2 and Figure 3), likely due to the maintenance of the SR and TT elements needed to allow Ca^2+^ entry (i.e., CEUs; Figure 4). As estrogen treatment also reduces oxidative stress, which is elevated in aging mice compared to younger controls (Figure 5), we also verified that administering NAC decreases the incidence of TAs in EDL muscles (Figure 6).

### 3.3. Effect of Estrogen Administration on Membrane Remodeling and SOCE Function

We hypothesized that the gender difference in the age-related accumulation of TAs in skeletal muscle [36,37,47] could be influenced by the sex hormone profile. Estrogen is a steroid hormone that is not only important for reproductive organs, but also exerts physiological effects on other organs and tissues, including skeletal muscle [60,61,62].

In the present paper, administration of exogenous 17-β-Estradiol to male mice (with extremely low endogenous levels) was able to significantly reduce the incidence of TAs in male animals (Figure 1), suggesting that endogenous estrogen may exert a protective role against the formation of TAs in female mice. Our data also point to a strict correlation between the reduced aggregation of TAs and the rescued SOCE function in treated mice. Indeed, EDL muscles from treated mice showed the ability to use external Ca^2+^ during repetitive stimulation, similar to that of EDLs from 10 mo. old mice (Figure 2 and Figure 3). This result creates a parallel with a previous article showing how a reduction in the aggregation of TAs in aged mice was also accompanied by an increase in SOCE function [46]. Dysfunctional SOCE in muscles containing TAs is probably the result of the improper accumulation of STIM1 and ORAI1 in TAs [46], while restoration of SOCE function is likely the result of the increased presence of CEUs. In this scenario, the increased presence of CEUs in exercised aged mice [46] and in mice treated with estrogen (Figure 4 of the present paper) seems to provide the structural framework for an improved coupling between STIM1 and ORAI1, hence an improved SOCE function.

### 3.4. Effect of Estrogen Administration on Oxidative Stress

There are several potential mechanisms underlying estrogen’s role as an antioxidant. First, estrogen, due to its 18-carbon atom backbone structure related in molecular composition to antioxidant compounds such as vitamin E, is thought to have high antioxidant properties by scavenging free radicals and stimulating the gene expression and activity of major antioxidant enzymes, thus mitigating the harmful effects of oxidative stress [63,64,65]. In addition, due to its structural similarity to cholesterol, estrogen may have the ability to intercalate into the phospholipid bilayer of the cell plasma membrane and increase membrane stabilization [66,67]. Finally, the discovery of three types of estrogen receptors has led to the hypothesis that estrogen may regulate a broad spectrum of downstream genes and molecular targets at the cellular level [68,69]. In skeletal muscle, both the two nuclear estrogen receptors α and β (ERα and ERb) and the plasma membrane G protein-coupled estrogen receptor (GPER) are expressed. ERα and ERb directly regulate cellular functions by regulating specific patterns of gene expression. GPER mediates rapid, non-genomic effects through membrane signaling pathways like cAMP, PI3K/Akt, and ERK/MAPK [70].

In a previous paper, we showed that nitration of protein tyrosine residues, as well as SOD-1 and SOD-2 expression, are both increased in the skeletal muscle of aged mice [71], and many authors also reported increased oxidative stress levels in sarcopenia [72,73,74,75,76]. Subsequent oxidative damage to proteins and membranes (which are critical for cellular function) could/would lead to improper Ca^2+^ handling and inefficient mitochondrial respiration, which could further exacerbate ROS production in a vicious feed-forward mechanism. We also demonstrated that regular/lifelong exercise: (a) successfully reduces the formation of TAs in aged mice; (b) prevents improper remodeling of membranes involved in EC coupling, specifically the junctional SR and the TT; and (c) prevents mitochondria uncoupling from EC coupling sites in muscles of aged mice and human subjects [46,77,78]. We propose that one of the possible mechanisms underlying damage during aging, as well as the protective effect of regular exercise, may involve oxidative stress levels, which increase due to aging but are reduced by regular exercise [77,78]. Oxidative stress levels are also critical in other pathological conditions; for instance, excessive production of ROS and RNS has been proposed to be a key step in the cascade of molecular events that leads to rhabdomyolysis of muscle fibers and the consequent death of malignant hyperthermia (MH) susceptible male mice [51,53,79]. We also demonstrated that: (d) treatment of MH susceptible mice with antioxidants (i.e., NAC) provides therapeutic benefits in reducing mitochondrial damage, limiting the development of structural core-like regions, and improving muscle function [51,52,53]. It was suggested that the different abilities of male and female animals to modulate oxidative stress were responsible for the sex-dependent MH susceptibility of male CASQ-1 knockout (or CASQ1-null) mice [80]. Indeed, in CASQ1-null mice, the presence of estrogen lowered levels of oxidative stress and provided effective protection against lethal MH-like events [80]. Finally, we also showed that aerobic training significantly reduced mortality in male CASQ1-null mice exposed to heat stress, enhancing endogenous antioxidant defenses and improving mitochondrial function [81].

Based on this evidence (and on the assumption that one of the potential mechanisms underlying estrogenic action within the cells involves modulation of the oxidative status), we treated male mice from 10 to 18 months of age with NAC 1%, a potent antioxidant, and verified the beneficial effect of NAC treatment in preventing the accumulation of TAs (i.e., reducing the number of fibers containing TAs; Figure 6E) and possibly inducing their fragmentation in smaller structures (suggested by the smaller size, without a reduction in the number; Figure 6F,G).

### 3.5. Final Remarks

Gender differences in the phenotypic expression of pathologies are becoming a prevalent topic in current research. In our experience, we became aware of this issue when we first realized how important gender was in studying the phenotype of CASQ1-knockout mice [79]. In CASQ1-null mice, males were more prone to spontaneous mortality and more susceptible to hyperthermic episodes in response to anesthetics, heat, and exertion [79,80]. Studying this mouse model, we then observed that these gender differences could arise from differences in oxidative stress [53,81] and that hormones might play a role in regulating oxidative stress levels [80]. Since TAs form only in the muscles of aging male mice, testing the effects of estrogen in males is a logical step. Indeed, estrogen administration proved itself to be effective in reducing the formation of TAs in male WT mice (Figure 1).

Because high oxidative stress levels (Figure 5) may contribute to TA formation, we also proved that NAC administration could reduce TA formation (Figure 6). This strategy was previously used to successfully reduce the formation of cores and susceptibility to malignant hyperthermia in mice carrying the human Y522S mutation in the RYR1 (YS mice) and in CASQ1-null mice [51,52,53]. From this perspective, oxidative stress appears to cause a cellular imbalance, contributing to multiple pathological mechanisms. In the hyperthermic phenotype, the mechanism underlying the lethal response to stressors involves nitrosylation of the ryanodine receptor [51].

Although one of the limitations of the study is that the mechanisms linking oxidative stress to the progressive assembly of TAs in sedentary WT mice remain unclear and warrant further investigation, the starting point is exercise [46] and estrogen administration (present paper), reduce both oxidative stress and the formation of TAs, while improving SOCE function.

Regarding the potential application of these findings to human sarcopenia or TA myopathies, given the multiple contraindications associated with estrogen therapy, the direct use of estrogens for treating TA myopathy seems unlikely at present. Nevertheless, evidence suggesting that estrogen may benefit muscle diseases like TA opens the door to identifying novel targets for the development of new therapies for this condition [82].

## 4. Materials and Methods

### 4.1. Animals

We studied different groups of male WT C57bl/6 mice: untreated 10 mo. and 18 mo. animals. These control and treated mice received drinking water containing a pharmacological treatment (see next paragraph) from 10 to 18 months of age. All in vivo experiments/protocols on animals were conducted according to the Directive of the European Union 2010/63/UE and the National Institutes of Health Guide for the Care and Use of Laboratory Animals and approved by the local Committee for Animal Care and Italian Ministry of Health (313/2019-PR; 15 April 2019). All mice were housed in microisolator cages at 20 °C in a 12 h light/dark cycle and provided with free access to water and food.

### 4.2. Pharmacological Treatment of Mice

(a) Treatment with 17-β-Estradiol (referred to as treated). 17-β-Estradiol was administered in drinking water ad libitum to male WT mice from 10 to 18 months of age. Because of estrogen’s low solubility in water, it was initially dissolved in 95% ethanol (5 mg/mL). Solubilized estrogen was subsequently administered to WT male mice in drinking water at a final dose of 440 ng/mL H_2_O, and it was changed twice a week. After 8 months of treatment (i.e., at 18 months of age), mice were killed by cervical dislocation as approved by the D. lgs n.26/2014, and EDL muscles were dissected for ex vivo analysis.
(b) Treatment with N-acetylcysteine (NAC 1%). The NAC group of male WT animals received ad libitum drinking water containing 1% weight/volume (1% *w*/*v*) of NAC from 10 to 18 months of age. This dose of NAC for 8 months did not cause any noticeable negative side effects to WT mice. After 8 months of treatment (i.e., at 18 months of age), mice were killed by cervical dislocation as approved by the D. lgs n.26/2014, and EDL muscles were dissected for ex vivo analysis.

### 4.3. Preparation of Samples for Histology and EM

EDL muscles from 10 mo., 18 mo., and estrogen/NAC 1%-treated mice were carefully dissected and fixed at room temperature (RT) in 3.5% glutaraldehyde in a 0.1 M sodium cacodylate (NaCaCO) buffer (pH 7.2), overnight. After primary fixation, the muscles were post-fixed in 2% OsO_4_ in NaCaCO buffer for 2 h and subsequently en block-stained in an aqueous saturated uranyl acetate replacement. For TT staining, samples were post-fixed in a mixture of 2% OsO_4_ and 0.8% potassium ferrocyanide [K_3_Fe(CN)_6_] for 1–2 h, followed by rinsing with 0.1 M NaCaCO buffer. After dehydration, specimens were embedded in an epoxy resin (Epon 812). For light microscopy, semithin (800 nm) sections were cut with a Leica Ultracut R Microtome (Leica Microsystem, Vienna, Austria) using a Diatome diamond knife (Diatome Ltd., Biel, Switzerland) and stained in a solution containing 1% Toludine blue O and 1% Sodium Borate Tetra in distilled water for 3 min on a hot plate at 55–60 °C. After washing and drying, sections were mounted using a mounting medium (DPX Mountant for Histology, SIGMA, St. Louis, MO, USA) and observed with a Leica DMLB light microscope (Leica Microsystem, Vienna, Austria).

For EM, ultrathin sections (50 nm) were cut using a Leica Ultracut R microtome (Leica Microsystem, Vienna, Austria) with a Diatome diamond knife (Diatome, Biel, Switzerland) and double-stained with uranyl acetate replacement and lead citrate. Sections were viewed in an FP 505 Morgagni Series 268D electron microscope (FEI Company, Brno, Czechia), equipped with a Megaview III digital camera and Soft Imaging System at 60 kV (Olympus Soft Imaging Solutions, Munster, Germany).

### 4.4. Quantitative Analysis

(a) Histology. For quantitative histological analysis, images were randomly collected from transversal sections of EDL fibers from 10 mo. (*n* = 3), 18 mo. (*n* = 3), and estrogen/NAC 1%-treated mice (*n* = 3). The percentage of fibers containing ordered arrays of SR tubes (i.e., tubular aggregates) was evaluated and reported as the mean value. Moreover, CSA of skeletal muscle fibers was measured on whole-muscle semithin transverse sections and plotted as the distribution frequency. In detail, histological analyses were conducted on three different EDL muscles (from three different mice) per experimental group. For each sample, multiple images were captured to cover the entire muscle cross-section. These images were then stitched together using the Leica DMLB optical microscope LAS software ver. 4.13.0 (Leica Microsystem, Vienna, Austria) to generate a single composite image representing the whole muscle section. Approximately 500 to 600 muscle fibers per sample were analyzed.

(b) EM. For all quantitative EM analyses, micrographs of non-overlapping regions were randomly collected from longitudinal and transverse sections of internal areas of EDL fibers from 10 mo. (*n* = 3), 18 mo. (*n* = 3), and estrogen/NAC 1%-treated (*n* = 3) mice. The following ultrastructural parameters were determined:

(c) Tubular aggregates (TAs). The number of TAs per fiber and the average size of TAs were evaluated and reported as the mean values, as shown by Boncompagni et al. 2021 [46].

(d) Sarcoplasmic reticulum (SR) stacks. Incidence of fibers exhibiting flat and long SR cisternae/tubes (expressed as percentage) and the number of SR stacks (reported as the average value in 100 μm^2^ for each section) were evaluated in micrographs collected from EDL muscle fibers in transverse sections, as shown by Boncompagni et al. 2017 [32]. In each specimen, 15–20 fibers were analyzed, and in each fiber, 5 micrographs were taken at 28,000× magnification.

(e) Non-triadic transverse tubule (TT) network at the I band. We determined both (i) the total network of the TT at the I band of the sarcomere and (ii) the extension of the SR in close association with the TT, as shown by Boncompagni et al. 2017 [32]. The TT network was evaluated in micrographs collected from EDL muscle fibers, either stained or not with ferrocyanide in transverse sections, and reported as the average length (micron) per area of the section (100 μm^2^). Operationally, TTs were identified by the presence of ferrocyanide black staining or the proximity of an originating triad. The measured distance obtained by drawing a line in correspondence with the TT and adjacent to an SR stack specifically corresponds to the TT/SR contact length. In each specimen, 15–20 fibers were analyzed, and in each fiber, 5 micrographs were taken at 28,000× magnification.

### 4.5. Ex Vivo Fatigue Protocol

Ex vivo measurements of the muscle’s contractile performance during repetitive high-frequency stimulation were carried out on intact EDL muscles of 10 mo. (*n* = 6), 18 mo. (*n* = 6), and 17-β-Estradiol-treated (*n* = 6) male mice. Muscles were carefully dissected from the hindlimbs and transferred to a dish containing a standard Krebs–Henseleit (KH) solution (18 mM NaCl, 5 mM KCl, 2.5 mM CaCl_2_, 1 mM KH_2_PO_4_,1 mM MgSO_4_, 25 mM NaHCO_3_, and 11 mM glucose, pH 7.4). Each muscle was pinned and secured at both ends using fine silk threads. Subsequently, muscles were vertically mounted in an organ bath filled with KH solution and placed between two platinum electrodes and attached to a servo motor and force transducer (model 1200A; Aurora Scientific, Aurora, ON, Canada). Optimal muscle length (L0) was determined by applying a series of 80 Hz stimulus strains every 1 min in order to adjust the muscle to the length that generated maximal force (F0), avoiding muscle fatigue. Twitch and tetanic contractile properties were then measured. Following these baseline measurements, EDL muscles were subjected to a repetitive high-frequency stimulation fatigue protocol consisting of 30 consecutive, 1 s duration, 60 Hz stimulus trains delivered every 5 s while being continuously perfused with KH solution. To assess the involvement of extracellular Ca^2+^ entry, additional experiments were performed under conditions designed to limit/block Ca^2+^ entry: (i) using nominally Ca^2+^-free KH solution (where external Ca^2+^ was replaced with an equimolar amount of Mg^2+^) and (ii) supplementing the standard KH solution with 10 mM BTP-2, a known SOCE inhibitor [83]. Prior to stimulation, muscles were equilibrated for at least 20 min in either Ca^2+^-free KH solution or the KH solution supplemented with BTP-2. Muscle force output was recorded using Dynamic Muscle Control software ver. 5.415 (Aurora Scientific) and analyzed using a combination of Dynamic Muscle Analysis ver. 5.200 (Aurora Scientific) software. Specific force (mN/mm^2^) was calculated by normalizing the absolute force (mN) to the physiological CSA (mm^2^) obtained as follows: wet weight (mg)/[L0 (mm) × 1.06 (mg/mm^3^) × 0.44] [33,34,84]. All experiments were conducted at RT.

### 4.6. Measurements of Oxidative Stress

EDL muscles were dissected from 10 mo., 18 mo., and 17-β-Estradiol-treated WT mice and quickly frozen in liquid nitrogen until further use. At the appropriate time, muscles were homogenized in RIPA buffer (TRIS HCl 50mM, pH 7.4, Triton-X 1%, Deoxycholate 0.25%, NaCl 150 mM, SDS 3%, EDTA 1 mM, and protease inhibitors 2.5%) using a mechanical homogenizer, and then centrifuged for 15 min at 900× *g* at 4 °C. Following supernatant collection, protein concentration was assessed using the BCA protein assay (ThermoFisher Scientific, Waltham, MA, USA). The same amount of total protein (20 μg) was resolved in 10–12% sodium dodecyl sulfate (SDS) polyacrylamide gel electrophoresis and transferred to nitrocellulose membranes. Blots were then blocked with 5% non-fat dry milk (EuroClone, Pero, Italy) in TBS-T 0.1% for 1 h. Membranes were incubated overnight at 4 °C with primary antibodies diluted in 5% non-fat dry milk in TBS-T, as follows: (a) anti-SOD-1 antibody (rabbit polyclonal 1:1000, Santa Cruz Biotechnology Inc., Dallas, TX, USA); (b) anti-SOD-2 antibody (rabbit polyclonal 1:1000, Santa Cruz Biotechnology, Inc., Dallas, TX, USA); and (c) anti-catalase antibody (rabbit polyclonal 1:1000, Santa Cruz Biotechnology Inc., Dallas, TX, USA). The mouse monoclonal anti-glyceraldehyde-3-phosphate dehydrogenase antibody (GAPDH) (1:10,000, TA802519, OriGene Technologies Inc., Rockville, MD, USA) was used as the loading control. After incubation with primary antibodies, membranes were washed 3 times for 10 min with TBS-T and then incubated for 1 h at room temperature with horseradish peroxidase–conjugated secondary antibodies (1:10,000, Merck Millipore, Burlington, MA, USA) diluted in 5% non-fat dry milk in TBS-T, followed by washes in TBS-T. Peroxidase activity was detected using enhanced chemiluminescent liquid (ECL, Perkin-Elmer, Waltham, MA, USA); the bands were visualized using a gel documentation system (UVItec Cambridge Ltd., Cambridge, UK); and the band densitometric quantification of signals was performed using the imaging system Alliance Mini 4 with Alliance 1D MAX software ver. 4.7/LD4 (UVItec Cambridge Ltd., Cambridge, UK).

### 4.7. Statistical Analysis

Statistical significance was determined using either GraphPad Prism ver. 6 (GraphPad Software, Boston, MA, USA) or Microsoft Excel ver. 365 for entrerprise (Microsoft Office, Redmond, WA, USA). One-way ANOVA tests and Student’s *t*-test were performed using GraphPad Prism ver. 6 software. In all cases, differences were considered statistically significant at *p* < 0.05. Data are shown as mean ± SEM.

## Data Availability

Dataset available on request from the authors.

## Abbreviations

The following abbreviations are used in this manuscript:

CEU	Ca^2+^ entry unit
EDL	Extensor digitorum longus
EM	Electron microscopy
LM	Light microscopy
NAC	N-acetylcysteine
SR	Sarcoplasmic reticulum
SOCE	Store-operated Ca^2+^ entry
STIM1	Stromal interaction molecule-1
TA	Tubular aggregate
TAM	TA myopathy
TT	Transverse tubule
WT	Wild type
